# Peptidoglycan binding protein (PGBP)-modified magnetic nanobeads for efficient magnetic capturing of *Staphylococcus aureus* associated with sepsis in blood

**DOI:** 10.1038/s41598-018-37194-2

**Published:** 2019-01-15

**Authors:** Jaewoo Lim, Jongmin Choi, Kyeonghye Guk, Seong Uk Son, Do Kyung Lee, Soo-Jin Yeom, Taejoon Kang, Juyeon Jung, Eun-Kyung Lim

**Affiliations:** 1 Hazards Monitoring Bionano Research Center, Korea Research Institute of Bioscience and Biotechnology (KRIBB), 125 Gwahak-ro, Yuseong-gu, Daejeon 34141 South Korea; 2 Department of Nanobiotechnology, KRIBB School of Biotechnology, University of Science and Technology (UST), 125 Gwahak-ro, Yuseong-gu, Daejeon 34113 Republic of Korea; 3 BioNano Health Guard Research Center, 125 Gwahak-ro, Yuseong-gu, Daejeon 34141 South Korea; 4 Synthetic Biology & Bioengineering Research Center, Korea Research Institute of Bioscience and Biotechnology (KRIBB), 125 Gwahak-ro, Yuseong-gu, Daejeon 34141 South Korea

## Abstract

Peptidoglycan-binding protein-modified magnetic nanobeads (PGBP-MNBs) were prepared for efficient magnetic capturing of Staphylococcus aureus (S. aureus), which is associated with sepsis, using the binding affinity of PGBP for the peptidoglycan (PG) layer on S. aureus. These PGBP-MNBs can simply capture S. aureus in plasma within 1 hr or even 15 min. Importantly, they also can capture various types of Gram-positive bacteria, such as Bacillus cereus and methicillin-resistant and methicillin-susceptible S. aureus (MRSA and MSSA). We believe that PGBP-based systems will be used to develop diagnostic systems for Gram-positive bacteria-related diseases.

## Introduction

*Staphylococcus aureus* (*S. aureus*) is a widely distributed Gram-positive pathogen that causes many serious diseases in humans, such as local purulent infections, pneumonia and sepsis. Some of these diseases are life-threatening clinical syndromes associated with significant patient morbidity and mortality^1–11^. Therefore, rapid and sensitive detection of *S. aureus* has become crucial for improving patient survival rates^12–17^. Because *S. aureus* is hard to detect at low concentrations (e.g., ≤100 CFU/mL), long reaction times are usually needed before analysis. The most commonly used methods are culture-based assays, which are often composed of a series of steps, including selective culture enrichment, differential plating, and biochemical/serological testing. However, these assays are slow, expensive, time consuming and labor intensive because the assay procedure is complicated and requires amplification or enrichment of *S. aureus* in the sample^18–21^. In this study, we developed a novel concentration platform functionalized with peptidoglycan-binding proteins (PGBPs) for expedient capturing and enrichment of Gram-positive *S. aureus*.

## Results and Discussion

**Figure 1:**
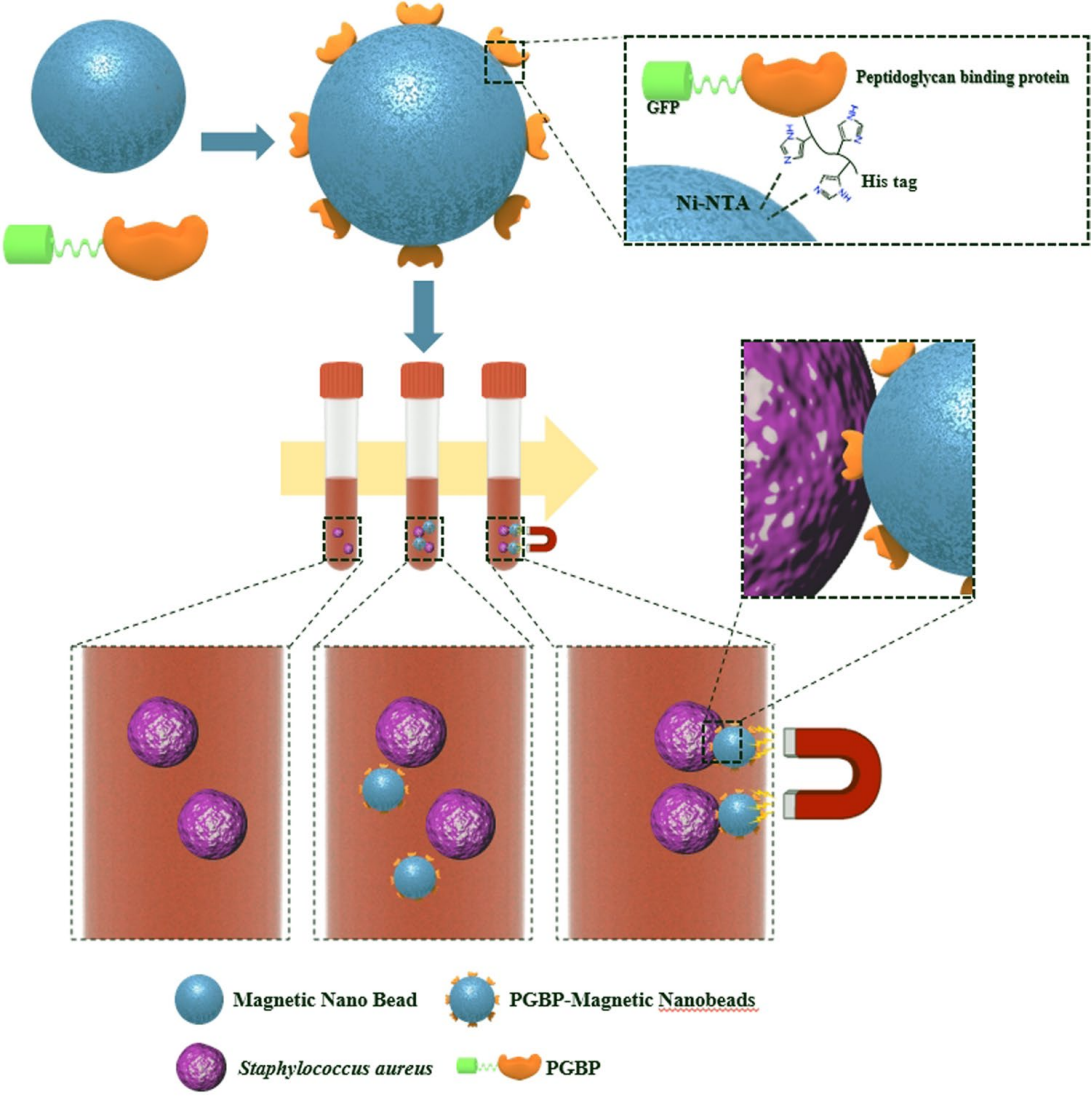
Preparation of peptidoglycan binding protein (PGBP)-modified magnetic nanobeads (PGBP-MNBs) for the efficient capturing of Gram-positive *Staphylococcus aureus* (*S. aureus*).

**Figure 2:**
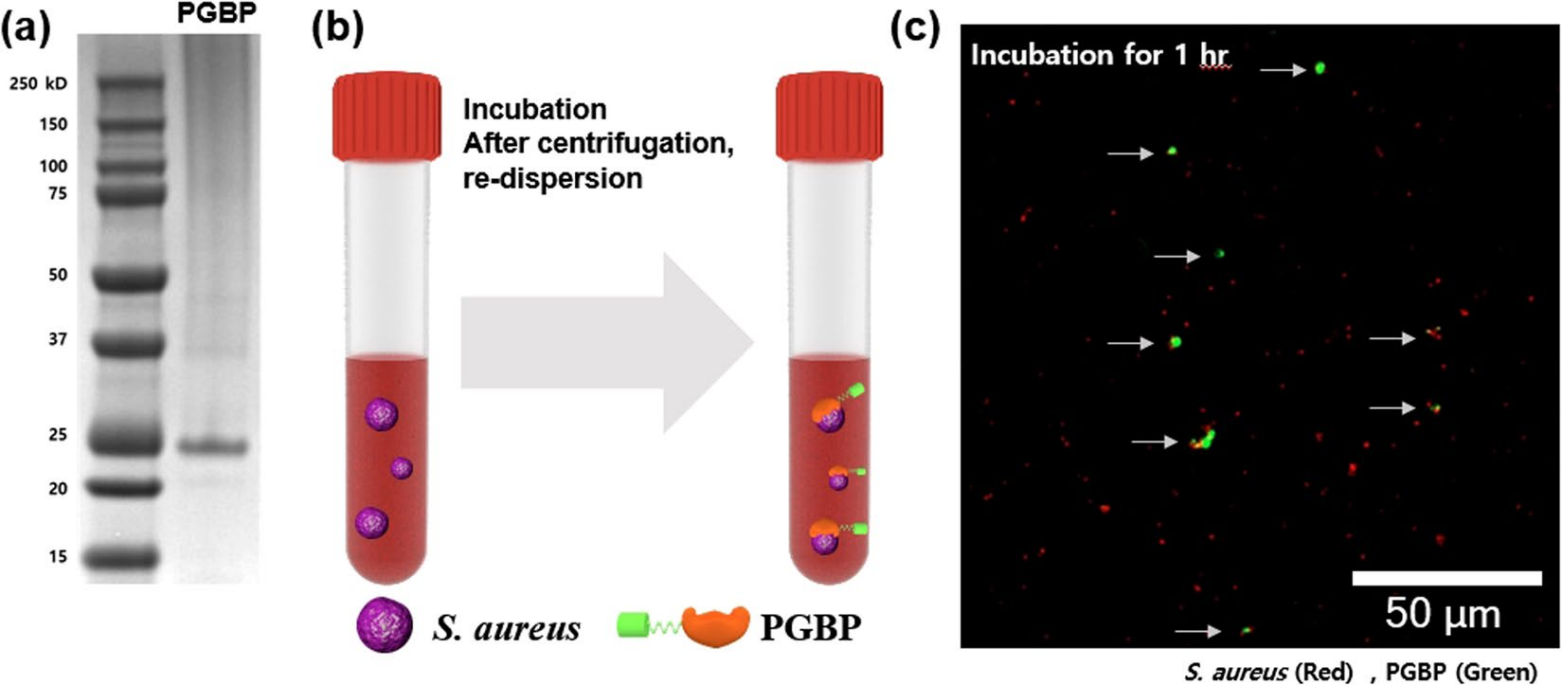
(**a**) The expression of PGBP analyzed by SDS-PAGE. (**b**) Schematic illustration of *S. aureus* in human plasma (10% plasma in PBS) captured by PGBP, and (**c**) its fluorescence image after co-incubation with *S. aureus* (Red) and PGBP (Green) for 1 hr at room temperature.

**Figure 3:**
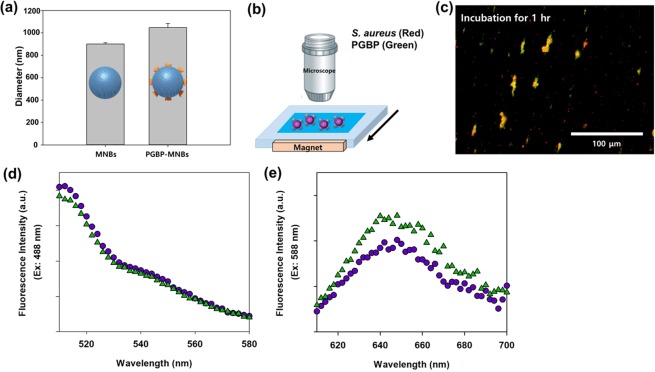
(**a**) Size measurement of MNBs and PGBP-MNBs by DLS analysis. (**b**) Illustration of magnetic separation of *S. aureus* using PGBP-MNBs. After binding with *S. aureus* and PGBP-MNBs for 1 hr, and (**c**) its fluorescence microscopic image (*S. aureus*: red and PGBP: green). Fluorescence spectra of PGBP-MNBs/*S. aureus* (**d**) at 488 nm (excitation) and (**e**) at 588 nm (excitation) under different reaction time (Circles: 1 hr and triangles: 2 hr), respectively.

**Figure 4:**
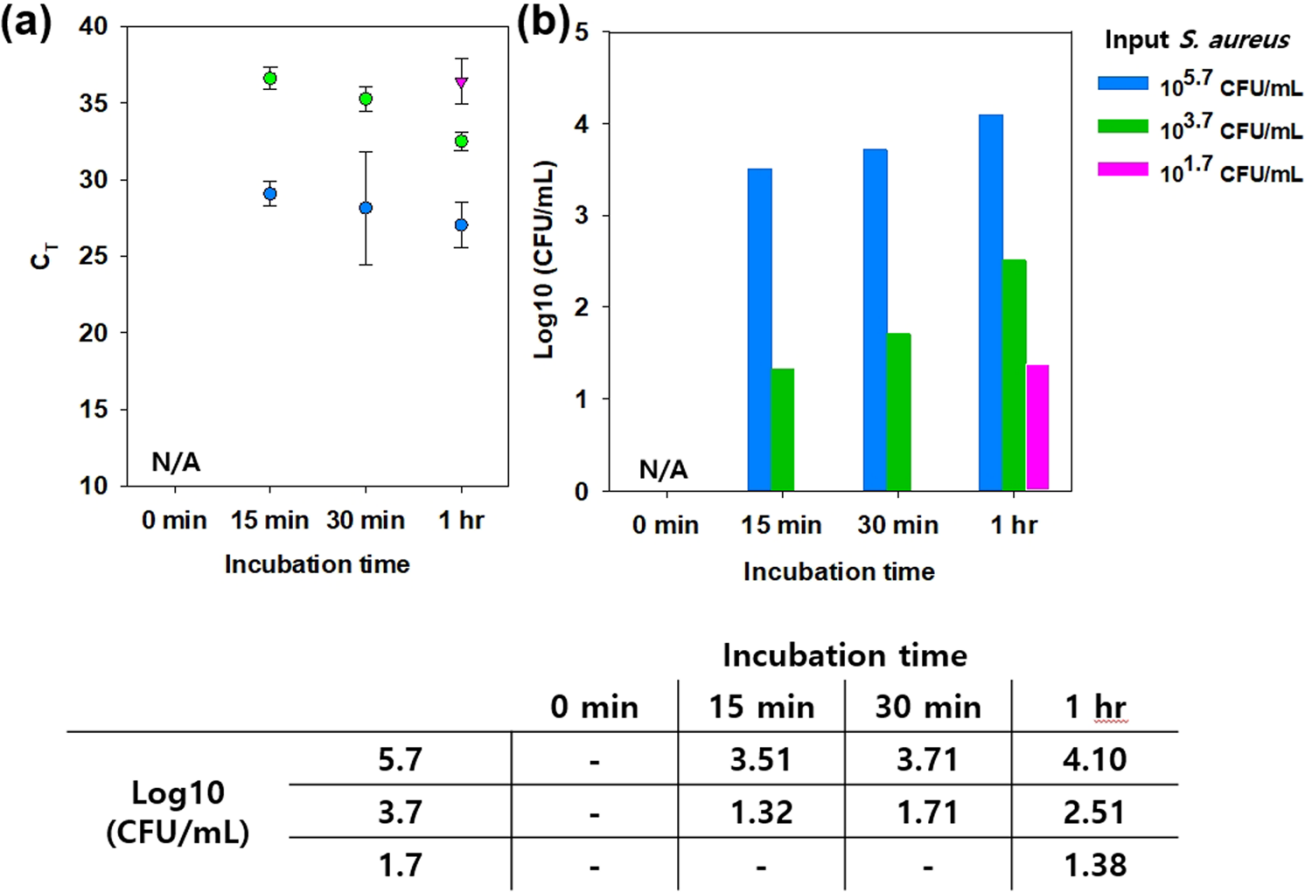
Real-time PCR analysis of *S. aureus* magnetically captured using PGBP-MNBs at various bacterial concentrations (Input: 10^5.7^, 10^3.7^ and 10^1.7^ CFU/mL) and reaction times (0 min, 15 min, 30 min and 1 hr). (**a**) Average threshold cycle value (Ct) of the captured *S. aureus* and (**b**) their corresponding output concentrations.

**Figure 5:**
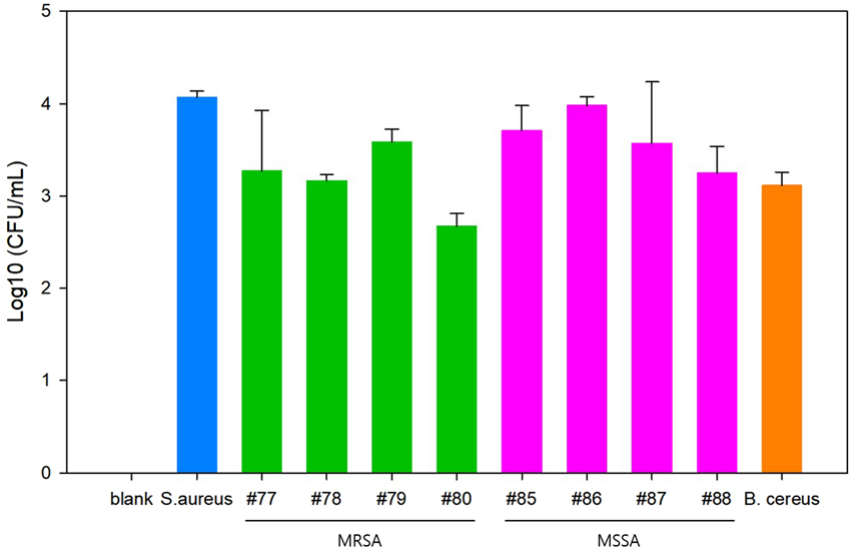
The concentrations of various types of bacteria (10^3.7^ CFU/mL) magnetically captured using PGBP-MNBs after 1 hr of incubation, as determined by real-time PCR (*S. aureus*: *Staphylococcus aureus*, *B. cereus*: *Bacillus cereus*, MSSA: methicillin-susceptible *Staphylococcus aureus* and MRSA: methicillin-resistant *Staphylococcus aureus*). Peptidoglycan-binding protein-modified magnetic nanobeads (PGBP-MNBs) were prepared for efficient magnetic capturing of *Staphylococcus aureus* (*S. aureus*), which is associated with sepsis, using the binding affinity of PGBP for the peptidoglycan (PG) layer on *S. aureus*.

The surface of the MNBs are modified with PGBP, enabling them to specifically recognize and strongly bind to the peptidoglycan (PG) layer on *S. aureus* (Fig. 1). PG is an essential component of the cell wall of all bacteria and is especially abundant in Gram-positive bacteria, in which it accounts for approximately half of the cell wall mass. It is well-known target for not only antibiotics but also the host immune response through recognition by pattern recognition receptors, including PGBPs^22–31^. Thus, PGBPs play a role in the recognition of PG in bacteria with nanomolar affinity. Thus, we developed MNBs modified with PGBP (PGBP-MNBs), and its PGBP is able to recognize *S. aureus* by binding to its PG layer like an antibody^32–34^. Furthermore, *S. aureus* captured by PGBP-MNBs was enriched by a magnetic field, allowing further analysis of whether a patient is infected with pathogens (*S. aureus*)^35–42^. We purified PGBP with a molecular weight of approximately 25 kDa, corresponding to approximately 421 amino acid. We also tagged PGBP with green fluorescent protein (GFP) to impart green fluorescence (Fig. 2a). The affinity of PGBP for the PG layer of Gram-positive *S. aureus* bacteria, which was measured as the binding affinity (K_D_), was determined using a BLItz system. The result showed that the K_D_ was 6.49 nM (Fig. S2). In addition, we visually confirmed the specific binding affinity of PGBP for PG layer from *S. aureus* by fluorescence microscopy analysis. *S. aureus* bacteria stained with red fluorescent reagents were incubated with PGBP (green fluorescence). After 1 hr, the bacteria were purified by centrifugation to eliminate unbound PGBP and then were re-suspended in buffer containing 10% plasma, similar to physiological blood conditions in humans (Fig. 2b). As shown in Fig. 2c, the red and green fluorescence signals were colocalized, indicating that PGBP was bound to *S. aureus*. It was confirmed in fluorescence spectra as well that PGBP/*S. aureus* showed both green and red fluorescence intensities (Fig. S3). PGBP-MNBs were prepared for magnetic capturing of *S. aureus* at room temperature. The bacteria were then enriched by applying a magnet, as Ni-NTA was immobilized on the MNBs along with PGBP^43–46^. Histidine (His)-tags on PGBP act as a chelating agent and form chelate complexes with nickel (Ni) ions from Ni-NTA, which offer vacant electron orbitals to form coordinate bonds. The size of the MNBs before PGBP binding was approximately 900 nm, and their size after PGBP binding increased to approximately 1 μm (Fig. 3a). After reaction of the PGBP-MNBs with *S. aureus* in buffer containing 10% plasma for 1 hr, the resulting PGBP-MNBs/*S. aureus* complex was dropped on a glass slide, and then a magnet was placed under this glass slide. The complex was magnetically manipulated by the external magnetic field, showing a yellow fluorescence signal overlapped with both the red and green fluorescence signals (Fig. 3b,c). In addition, we recorded fluorescence spectra of the PGBP-MNBs/*S. aureus* complex at excitation wavelengths of 488 and 588 nm using a multimode-microplate reader to confirm the binding capacity of PGBP-MNB for *S. aureus*. Free PGBP, MNBs and *S. aureus* were also measured in the same manner as controls (Fig. S3). The PGBP-MNB/*S. aureus* complex showed fluorescence intensities corresponding GFP, which is similar to free PGBP (Figs 3d and S3). In addition, at the excitation wavelength of 588 nm, PGBP-MNBs/*S. aureus* exhibited fluorescence signals corresponding to *S. aureus*. As expected, as the reaction time increased, the fluorescence intensity under 588 nm excitation increased, indicating that the amount of *S. aureus* captured by the PGBP-MNBs was increased (Figs 3e and S3). Therefore, the ability of PGBP-MNBs to capture *S. aureus* could increase with the reaction time. Furthermore, for quick acquisition of *S. aureus* by magnetic concentration using these PGBP-MNBs, we evaluated the capturing abilities of *S. aureus* by the PGBP-MNBs with different reaction time and *S. aureus* concentrations by real-time PCR. First, *S. aureus* bacteria at concentrations of 10^5.7^, 10^3.7^ and 10^1.7^ CFU/mL (according to the optical density at 600 nm) were incubated with PGBP-MNBs for various reaction times (0 min, 15 min, 30 min and 1 hr). Subsequently, the PGBP-MNBs magnetically captured *S. aureus* by forming PGBP-MNBs/*S. aureus* complexes, and Ct values and concentrations of the captured *S. aureus* were measured using real-time PCR^46–50^. As mentioned above, the longer the reaction time was, the higher the capturing ability (Fig. 4). After the PGBP-MNBs were mixed with *S. aureus* for 1 hr, a point magnet easily attracted *S. aureus* bacteria because their cell surfaces were bound to the PGBP-MNBs. Even at a *S. aureus* input concentration of 10^1.7^ CFU/mL, approximately 10^1.38^ CFU/mL *S. aureus* bacteria were captured by the PGBP-MNBs (Fig. 4b). Importantly, the PGBP-MNBs still showed sufficient ability as capturing probes at a short reaction time of 15 min. We further evaluated whether the PGBP-MNBs were capable of detecting Gram-positive bacteria regardless of species (Fig. 5). We chose *S. aureus*, *Bacillus cereus*, methicillin-susceptible *Staphylococcus aureus* (MSSA) and methicillin-resistant *Staphylococcus aureus* (MRSA) as Gram-positive bacteria with a thick PG layer as Gram-positive bacteria^18,51,52^. Notably, MRSA is one of the most common resistant *S. aureus* strains in hospitals. *S. aureus* and *B. cereus* were obtained from the Korean Collection for Type Cultures (KCTC), and clinically isolated MRSA (#77, #78, #79 and #80) and MSSA (#85, #86, #87 and #88) were obtained from BioNano Health Guard Research Center (H-GUARD). Bacteria (10^3.7^ CFU/mL) were separately mixed with PGBP-MNBs for 1 hr at room temperature. After incubation, the unbound bacteria were removed by magnetically assisted washing, and then the captured bacterial concentrations were measured by real-time PCR assay. The data revealed the great efficacy of the PGBP-MNBs in magnetic capturing of approximately 10^2.8^~10^4^ CFU/mL Gram-positive bacteria with a capture efficiency of about 81.67%^53^. These results confirmed that PGBP-MNBs could be used universally to efficiently detect most Gram-positive bacteria.

## Conclusions

We developed PGBP-MNB as a magnetic capturing probe of Gram-positive *S. aureus*, which is associated with sepsis, and confirmed its ability to capture these bacteria within just 15 min at room temperature. In particular, since there is a PG layer on Gram-positive bacteria, the PGBP-MNBs can capture not only *S. aureus* but also *B. cereus*, MRSA and MSSA. Notably, because it uses a protein (PGBP) that binds universally to Gram-positive bacteria, this probe (PGBP-MNBs) has increased practicality compared to probes that increase selectivity by using an antibody that binds to a specific bacterium. Furthermore, we expect this PGBP-based capture platform to be applicable to diagnostic systems for bacteria-related diseases.

## Experimental Section

### Chemicals

We purchased HisPur™ Ni-NTA Magnetic Beads and BacLight Red Bacterial Stain from THERMO FISHER SCIENTIFIC (USA). SYBR Green PCR master mix was purchased from QIAGEN (Germany). Dulbecco’s phosphate buffered saline (DPBS, 1X) was purchased from GIBCO (Life Technologies). Human plasma (pooled normal, K2 EDTA) was purchased from INNOVATIVE RESEARCH INC. (USA). A Mini-BEST bacterial genomic DNA extraction kit was purchased from TAKARA (Japan). Primer sets for bacterial DNA amplification by real-time PCR were purchased from BIONEER INC. (Korea), and their sequences are summarized in Table S1.

### Cloning, expression and purification of PGBP

We constructed a green fluorescence protein (GFP)-tagged peptidoglycan binding protein (PGBP) vector to express the fusion protein of PGBP and GFP (Fig. S1). The prepared expression vector was transformed into BL21 (DE3) component *Escherichia coli* expressing the recombinant protein, and the obtained transformant was inoculated into LB liquid medium supplemented with 50 μg/mL of ampicillin and then cultured at 37 °C until the optical density (OD) at 600 nm reached 0.6. After adding 1 mM isopropyl β-D-1-thiogalactopyranoside (IPTG), the cells were further cultured with shaking for 4 hr to obtain the recombinant protein (PGBP). To extract the expressed recombinant protein, 20 mM Tris-Cl (pH 8.0) and 0.2 M NaCl buffer solution was added to the *E. coli* cells, which were then recovered by centrifugation to suspend the cells and lysed using an ultrasonicator. The lysate was dissolved in an 8 M urea solution for efficient refolding of the insoluble protein, was subjected to metal affinity chromatography using 6X histidine as a metal affinity tag and was dialyzed against refolding solution (50 mM Tris-HCl (pH 8.5), 1 M arginine, 2 mM EDTA, 5 mM cysteamine, and 0.5 mM cystamine) at 4 °C for 48 hr under stirring. After sufficient refolding, the buffer of the recombinant protein was exchanged with PBS (pH 7.4) using ultrafiltration (MWCO: 10 KDa) and was concentrated to 1 mg/mL.

### Bacterial culture and harvest conditions

The bacterial strains of *Staphylococcus aureus* (KCTC: 1621), *Bacillus cereus* (KCTC: 3624), methicillin-resistant *Staphylococcus aureus* (MRSA) (#77, #78, #79, and #80) and methicillin-susceptible *Staphylococcus aureus* (MSSA) (#85, #86, #87, and #88) were supplied by the Korean Collection for Type Cultures (KCTC), KRIBB (Korea) and BioNano Health Guard Research Center (H-GUARD) (Korea). All bacterial stains were cultured with LB broth (USA) at 37 °C. The bacterial concentration of *S. aureus* was determined by measuring the optical density (OD) at 600 nm (an OD600 of 1.0 Au = 8.8 × 10^8^ CFU/mL).

### Binding capacity of PGBP for *S. aureus* (PGBP/*S. aureus*)

We measured the binding affinity between PGBP and *S. aureus* using a biolayer interferometry-based biosensor BLItz system (FORTEBIO). PG from *S. aureus* was purchased from SIGMA-ALDRICH. PGBP loaded on Ni-NTA biosensors to bind with 6X histidine of PGBP and Ni-NTA, equilibrated in PBS for 1 min to establish a stable baseline, and then dipped into 4uL of PG from *S. aureus* (0 ~ 50 nM) to obtain the association curve for 300 s. The dissociation curve was obtained for 300 s using dipping holder. Afterwards, Binding affinities were calculated by fitting the curves using BLItz software (Fig. S2). We also performed binding capacity of PGBP against *S. aureus* in a same manner. Moreover, to visually confirm the binding of PGBP for *S. aureus* (10^7^ CFU/mL), we used a red fluorescent dye (BACLIGHT RED BACTERIAL STAIN) for bacterial staining (Ex: 581–596 nm/Em: 644 nm) (THERMO FISHER SCIENTIFIC). Then, bacteria were co-incubated with PGBP (1 mg/mL, 20 µL) for 1 or 2 hr at 37 °C. After incubation, unbound PGBPs were washed with PBS three times by centrifugation at 10,000 rpm for 10 min. The PGBP/*S. aureus* complex was observed by Jaewoo Lim using a fluorescent Microscope (EVOS Cell Imaging Systems, THERMO FISHER SCIENTIFIC).

### Preparation of PGBP-magnetic nanobeads (PGBP-MNBs)

Ni-NTA magnetic beads (8 µL, 12.5 mg/mL) and 10 µL of PGBP (1 mg/mL) (the ratio of magnetic beads to PGBP was 1:0.1) were mixed in PBS (1 mL) and incubated overnight at 4 °C. Then, unbound PGBP was removed from the PGBP-MNBs by washing three times with PBS using a magnetic tube rack, and the PGBP-MNBs were redispersed in PBS. The PGBP-MNBs were stored at −20 °C before use.

### Ability of PGBP-MNBs to capture *S. aureus* (PGBP-MNBs/*S. aureus*)

We prepared 10^1.7^~10^5.7^ CFU/mL *S. aureus* in human plasma solution (10% human plasma in PBS). Then, 1 mL of PGBP-MNBs (0.1 mg/mL) were injected into the bacterial sample, and then they (PGBP-MNBs/*S. aureus*) mixed for 15 min, 30 min and 1 hr, respectively. To remove unbound bacteria, the samples were washed three times with PBS using magnetic separation methods. Additionally, we confirmed the capturing ability using various Gram-positive bacteria, including *S. aureus, B. cereus*, MRSA and MSSA. In these experiments, each type of bacteria (10^3.7^ CFU/mL) was separately mixed with the PGBP-MNBs (1 mL, 0.1 mg/mL) for 1 hr, and then unbound bacteria were removed by using magnetic separation methods.

### Confirmation of the capture of *S. aureus* captured by PGBP-MNBs (PGBP-MNBs/*S. aureus*)

To confirm the capture ability of the PGBP-MNBs, we conducted flow cytometry analysis and real-time PCR. Flow cytometry analysis was performed using a FACSCalibur instrument (BECTON DICKINSON AND CO., USA) and software (WINMD) for data analysis. PGBP was tagged with GFP to impart green fluorescence, and *S. aureus* was stained with a red fluorescence dye. In addition, we carried out real-time PCR using a CFX96 Touch™ real-time PCR detection system (BIO-RAD LABORATORIES, USA) to measure the concentration of *S. aureus* captured by the PGBP-MNBs (PGBP-MNBs/*S. aureus*). The primer sets for amplification of *S. aureus* genomic DNA and control (16s rRNA) primer sequences were described in previous papers. PCR conditions were in accordance with the product manual of QuantiTect SYBR Green PCR kits (QIAGEN, Germany). We also measured the concentrations of the other types of captured bacteria (*B. cereus*, MRSA and MSSA) by real-time PCR in the same manner.

## Supplementary information

Supplementary information
