# Supplementary material for: Peptidoglycan binding protein (PGBP)-modified magnetic nanobeads for efficient magnetic capturing of Staphylococcus aureus associated with sepsis in blood
Source: Sci Rep. 2019 Jan 15;9:129. doi: 10.1038/s41598-018-37194-2 (PMC6333782; doi:10.1038/s41598-018-37194-2)
Supplement: Supplementary file 1 — Supplementary information [file 41598_2018_37194_MOESM1_ESM.docx]

Peptidoglycan binding protein (PGBP)-modified magnetic nanobeads for efficient magnetic capturing of *Staphylococcus aureus* associated with sepsis in blood

Jaewoo Lim^a, b^, Jongmin Choi^c^, Kyeonghye Guk^a,b^, Seong Uk Son^a^, Do Kyung Lee^c^, Soo-Jin Yeom^d^, Taejoon Kang^a,b^, Juyeon Jung^a, b*^ and Eun-Kyung Lim^a, b*^

^a^Hazards Monitoring Bionano Research Center, Korea Research Institute of Bioscience and Biotechnology (KRIBB), 125 Gwahak-ro, Yuseong-gu, Daejeon, 34141, South Korea

^b^Department of Nanobiotechnology, KRIBB School of Biotechnology, University of Science and Technology (UST), 125 Gwahak-ro, Yuseong-gu, Daejeon, 34113, Republic of Korea

^c^BioNano Health Guard Research Center, 125 Gwahak-ro, Yuseong-gu, Daejeon, 34141, South Korea

^d^Synthetic Biology & Bioengineering Research Center, Korea Research Institute of Bioscience and Biotechnology (KRIBB), 125 Gwahak-ro, Yuseong-gu, Daejeon, 34141, South Korea

^*^Correspondence and requests for materials should be addressed to E-K Lim (email: eklim1112@kribb.re.kr)


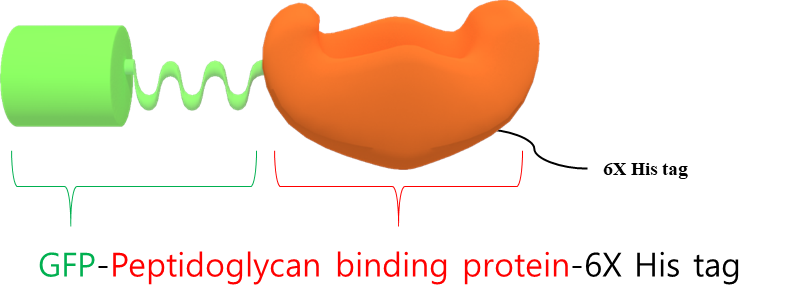


MVSKGEELFTGVVPILVELDGDVNGHKFSVSGEGEGDATYGKLTLKFICTTGKLPVPWPTLVTTLTYGVQCFSRYPDHMKQHDFFKSAMPEGYVQERTIFFKDDGNYKTRAEVKFEGDTLVNRIELKGIDFKEDGNILGHKLEYNYNSHNVYIMADKQKNGIKVNFKIRHNIEDGSVQLADHYQQNTPIGDGPVLLPDNHYLSTQSALSKDPNEKRDHMVLLEFVTAAGITLGMDELYKGGSGSGEFMVCPNIIKRSAWEARETHCPKMNLPAKYVIIIHTAGTSCTVSTDCQTVVRNIQSFHMDTRNFCDIGYHFLVGQDGGVYEGVGWHIQGSHTYGFNDIALGIAFIGYFVEKPPNAAALEAAQDLIQCAVVEGYLTPNYLLMGHSDVVNILSPGQALYNIISTWPHFKHLEHHHHHH

**Figure S1.** Sequence of PGBP, composed of green fluorescence protein (GFP)-peptidoglycan binding protein-6X His tags (GFP: Green, peptidoglycan binding proteins: Red and 6X Histidine: Black).


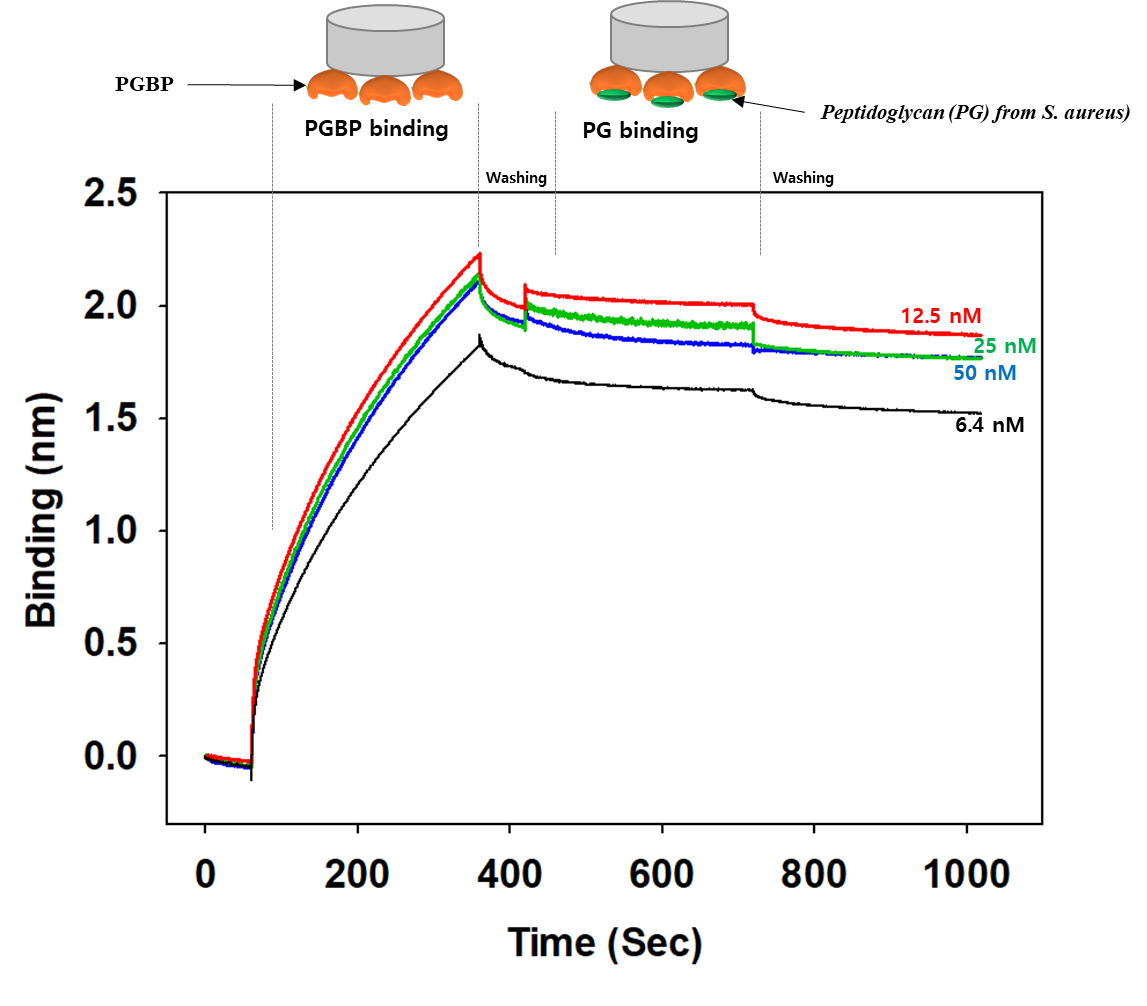


**Figure S2.** Measurement of binding affinity of PGBP with PG (6.4 ~ 50 nM) from *S. aureus* using the BLItz^®^ system.


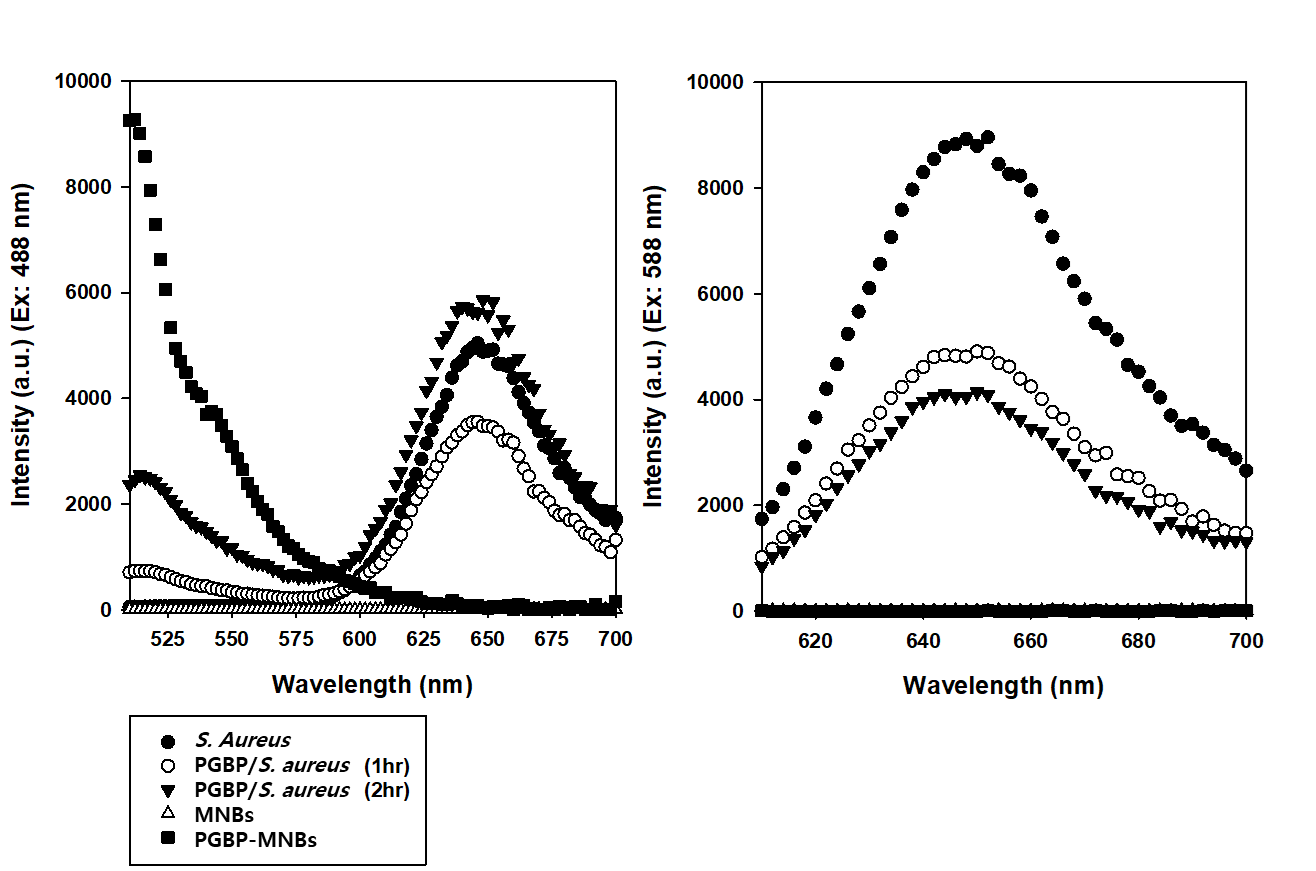


**Figure S3.** Fluorescence spectra of free *S. aureus*, MNBs, PGBP-MNBs and PGBP/*S. aureus* (b) at 488 nm (excitation) and (c) at 588 nm (excitation) under different incubation time (1 and 2 hr), respectively.


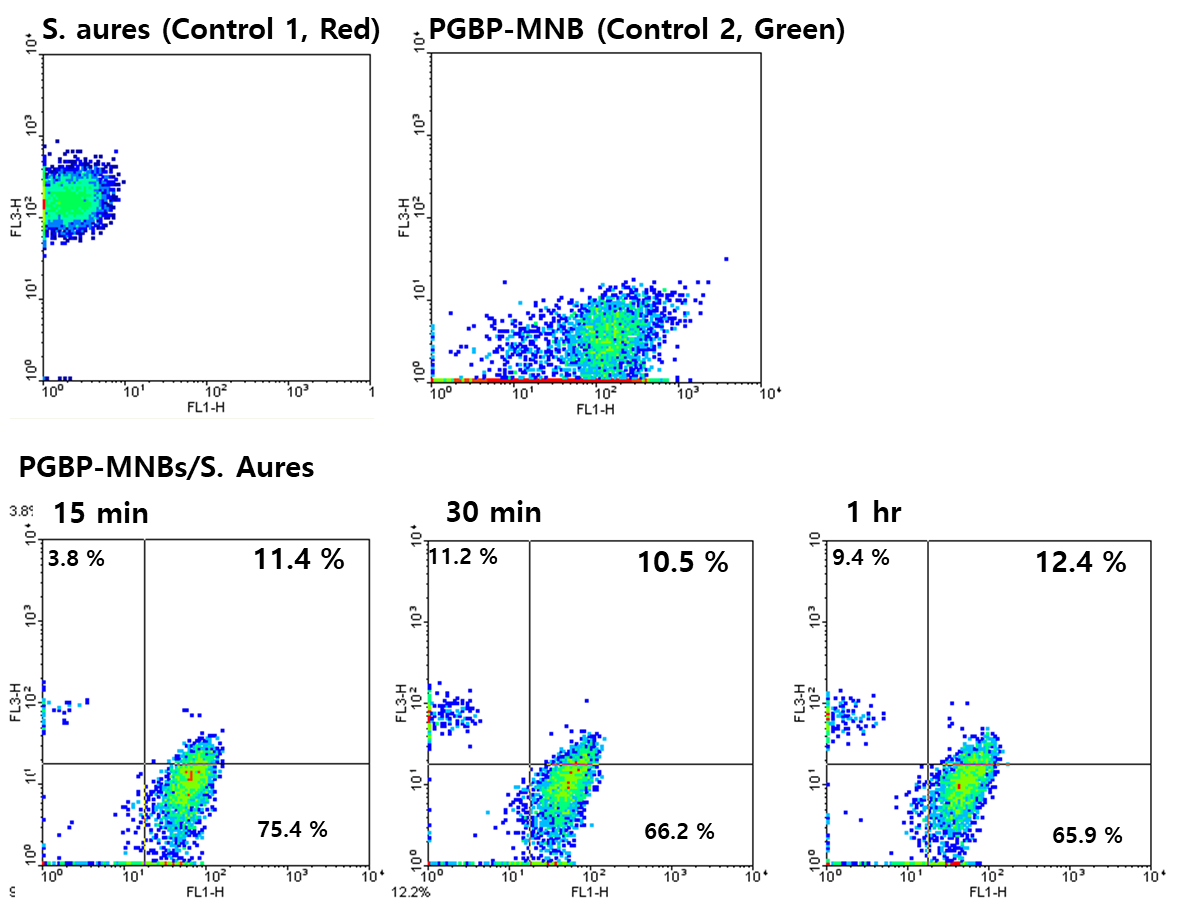


**Figure S4.** The magnetic capturing efficiency of *S. aureus* using PGBP-MNBs under various reaction time (15 min, 30 min, and 1 hr) by flow cytometry. Free *S. aureus* and PGBP-MNBs are used as control, respectively (FL1-H filter: Green and FL3 filter: Red).


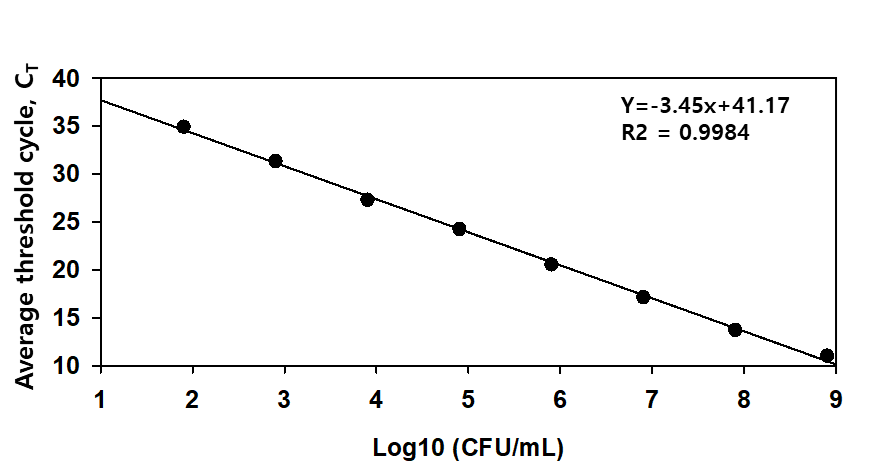


**Figure S5.** Linear regression of 10-fold dilutions of *S. Aureus* strain against corresponding average cycle threshold values (C_T_). Data points represent the means of three separate real-time PCR.

| Target | Primers | Sequences (5’-3’) |
| --- | --- | --- |
| *S. aureus*,  MRSA, MSSA | Forward | CCTGAAGCAAGTGCATTTACGA |
|  | Reverse | CTTTAGCCAAGCCTTGACGAACT |
| *B. cereus* | Forward | CTGTAGCGAATCGTACGTATC |
|  | Reverse | TACTGCTCCAGCCACATTAC |

**Table S1.** Sequences of primers used for identification of bacteria by real-time PCR
